# Review of prescribing information for influenza vaccines for pregnant and lactating women

**DOI:** 10.1016/j.vaccine.2016.08.042

**Published:** 2016-08-29

**Authors:** Tina Proveaux, Philipp Lambach, Justin R. Ortiz, Joachim Hombach, Neal A. Halsey

**Affiliations:** a Institute for Vaccine Safety, Johns Hopkins Bloomberg School of Public Health, 615 N. Wolfe Street, Baltimore, MD 21205, USA; b Initiative for Vaccine Research, World Health Organization, 20 Avenue Appia, 1211 Geneva 27, Switzerland

**Keywords:** Influenza vaccine, Pregnancy, Lactation, Vaccine safety

## Abstract

Information provided by most influenza vaccine manufacturers do not reflect the recommendations of WHO and/or national public health advisory groups with regard to the use of influenza vaccines in pregnant or lactating women. The majority of vaccines contain precautionary language which could discourage use in pregnant women and some include stronger language discouraging or contradicting use in pregnant or lactating women. Regulators and manufacturers should regularly assess the language of pregnancy and lactation sections in product information for vaccines and include information from national public health advisory groups regarding use by pregnant or lactating women and data from relevant studies.

## 1. Introduction

The World Health Organization, the U.S. Centers for Disease Control and Prevention [1], and the Public Health Agency of Canada [2] recommend that pregnant women be given high priority for seasonal influenza vaccination. Maternal influenza immunization has the potential to reduce disease during early childhood by preventing disease in newborns through trans-placental transfer of antibodies [3]. Based on the substantial risk of severe disease in neonates and the safety of influenza vaccine during pregnancy, the WHO recommends that pregnant women should be immunized at any stage during pregnancy [4,5]. In 2014, the WHO Global Advisory Committee on Vaccine Safety, in a safety review of inactivated influenza vaccine given during pregnancy, found no safety signals, although most of the data was from vaccination in the second and third trimester [6]. Several recent systematic reviews re-confirmed these findings of no safety signals [7–9]. Most of the available data are from studies of women who were known to be pregnant at the time of vaccination. One unpublished study in the United States has suggested a possible signal of increased spontaneous abortion when vaccine was administered very early in pregnancy [10]. Regulatory authorities conduct product specific reviews and they usually require data on the individual products to support package product information. National and international technical advisory groups often make recommendations considering all products, such as inactivated influenza vaccines, to be similar unless there are data indicating otherwise. Additional studies are indicated with different products where data are lacking, but the abundance of evidence clearly indicates a substantial benefit from multiple inactivated influenza vaccines for women who are known to be pregnant. In spite of recommendations for pregnant women to receive influenza vaccines, maternal influenza immunization rates remain low in many countries [4].

Identification of barriers to influenza vaccine uptake is relevant for policy makers who wish to increase acceptance of vaccines in pregnant women. Health care providers can affect use in pregnant women who are cautious about vaccinations because they are assuming responsibility for their life and that of their fetus [11,12]. As pregnancy is often an exclusion criterion in clinical trials, the availability of adequate and well-controlled, product specific clinical data is limited [13] and safety information for influenza vaccines is mainly based on observational study and post-licensure surveillance data [14]. Language in vaccine product information is therefore formulated based on precautionary principles by regulators and pharmaceutical companies [15]. Overly cautionary and/or restrictive language in vaccine information regarding use in pregnant and/or lactating women could be an obstacle to the use of influenza vaccine. The use of standardized fact-based information is critical to avoid misperceptions in the various audiences reading the product information. The objective of this review was to determine the degree of variability of the wording of influenza vaccine product information for descriptions of use by pregnant and lactating women.

Product information in the European Union is called Summary of Product Characteristics and generally has detailed information to the prescriber. Package Inserts are the United States equivalent and typically contain detailed safety information from clinical trials [16]. We use “product information” to describe this material provided by the manufacturer and approved by the regulator regarding use of influenza vaccines, including package inserts, package labels, package leaflets, patient information labeling, product description and summary of patient characteristics.

## 2. Methods

In a previous review [17] we identified 108 influenza vaccines marketed in recent years. Types of influenza vaccines recently produced include live and inactivated, monovalent, bivalent, trivalent, or quadrivalent preparations, whole virion, split-virus (with or without adjuvants), surface antigen, and virosomal. We identified the vaccines where product information was still available online. We also searched online through the Food and Drug Administration (www.fda.gov), the European Medicines Agency site (www.ema.europa.eu/ema), and the widely used internet search engine, Google (www.google.com) for product information for influenza vaccines. We attempted to find the most recent product information for seasonal, pandemic and pre-pandemic influenza vaccines. When information was found for the same vaccine produced in different years, only information for the most recently produced vaccine was included. Vaccines marketed under different names in different countries were listed under each different name.

We identified products with information written in English and available online. The product information was searched for the words “pregnant”, “pregnancy”, “lactating” and “lactation” for pertinent information regarding the use of the vaccine in pregnant and/or lactating women. Information was identified for influenza vaccines in other languages but we did not conduct searches in other languages. For example, some influenza vaccine manufacturer websites were available with information translated into English but offered only non-English product information. There are likely additional products with information in countries with no English language information available. For vaccines with product information in a language other than English, we used Google translate (translate.google.com) to translate the words “pregnant”, “pregnancy”, “lactating” and “lactation” from English to the language of the prescribing information and then searched the product information for pertinent text regarding the use of the vaccine in pregnant and/or lactating women. We also translated the word “age” from English to find information regarding approved ages for use.

## 3. Results

### 3.1. Information on use during pregnancy

We identified product information for 96 influenza vaccines; 31 pre- or pandemic (H1N1 and H5N1) and 65 seasonal vaccines (Table 1). Twenty vaccines (21%) had language suggesting that, when considering for use in pregnant women, official recommendations should be taken into account; 16 were in product information for pandemic influenza vaccines and 4 were for seasonal vaccines.

One-half (48) of the vaccines had product information with language suggesting that users consult a health care provider to determine whether the vaccine should be administered during pregnancy. The next most common category (27%) was vaccines with information suggesting use “if clearly needed” without further explanation of “clearly needed”. Two vaccines that had information recommending that a health care provider assess the situation also noted that people in high risk groups should receive the vaccine at any stage of pregnancy.

Only 10 of the vaccines had product information that suggested use during pregnancy: one from Australia, one from Canada, one from East Asia, and the remaining 7 were from EU countries. Among these 10 vaccines, there was a wide variety of information regarding safety data and what stage of pregnancy was preferred. One vaccine’s information indicated that influenza vaccines can be used throughout pregnancy. Seven vaccines included product information stating that inactivated influenza vaccines can be used in all stages of pregnancy and included statements that safety data from worldwide use do not show attributable adverse events in fetuses or pregnant women and that there are more studies available from use in the second and third trimester than first trimester. Information for one vaccine suggested use after the first trimester. Product information from another vaccine recommends influenza vaccine “to pregnant women who will be in the second or third trimester during the influenza season, including those in the first trimester at the time of vaccination” and further indicates that the vaccine has not been evaluated in pregnant women. Product information from another vaccine suggests that unadjuvanted vaccine has a long history of safe use in pregnant women and is therefore preferred, but that the specific vaccine has not been studied in pregnant women.

Product information from 4 vaccines indicated that influenza vaccine should not be used in pregnant women: 2 were formulations licensed in some EU countries [one is no longer authorized for use], the others were used in two large developing countries. The wording of one of the EU country vaccine product information indicated that it was ‘not recommended during pregnancy’. Among the developing country vaccines, one was ‘not recommended to pregnant or lactating women’, the other lists ‘women during pregnancy’ among the groups who are forbidden to use the product.

At the time of this study, 19 of the 96 vaccines we identified were approved for use in the United States and all included wording indicating that the vaccine ‘should be given to a pregnant woman only if clearly needed’, including 11 vaccines indicating Pregnancy Category B (animal studies have been performed) and 8 vaccines indicating Pregnancy Category C (no animal studies have been conducted). The US FDA is changing the categories, effective June 30, 2015 [18], and future package inserts will include information regarding infertility and pregnancy registries; it is unclear how these changes will affect the product information for use in pregnancy and lactation reported here.

The wording in package information differed for seasonal and pandemic vaccines. Of the 65 seasonal vaccines, 9 (14%) indicated the vaccines could be used in pregnancy, 29 (45%) indicated use if a consulted health care provider recommends the vaccine and 17 (26%) indicated ‘if clearly needed’.

Of the pre- and pandemic vaccines, 19/31 (61%) indicated they were recommended for use in pregnancy if a health care provider decided, 9 (29%) indicated ‘if clearly needed’. Recommendations against use by pregnant women were found for only 1 of the 31 pre-pandemic vaccines.

### 3.2. Information on use during breastfeeding

We found information concerning vaccination of lactating women for 90 of the 96 influenza vaccines (information for 6 vaccines did not mention breastfeeding or lactation) (Table 2). Nearly half of the product information indicated that influenza vaccine can be used by breastfeeding mothers. Only four vaccines (all were for pandemic influenza) had language suggesting that, when considering for use in lactating women, official recommendations should be taken into account.

## 4. Discussion

Despite the favorable risk-benefit profile for influenza vaccination of pregnant and lactating women recommended by WHO, many governments, obstetricians, and expert groups [4,5,19], product information for some vaccines limit or even contraindicate the use of influenza vaccines in pregnant women. Contraindications are usually given only if the risk to pregnancy or the developing fetus significantly outweighs the potential benefit to the mother or the fetus [19]. Adding contraindications to product information not only prohibits the vaccine’s use in the country of manufacture, but has discouraging effects on importing countries where favorable vaccination policies are in place.

Misperceptions and lack of awareness regarding influenza vaccine efficacy and safety have been identified as barriers to vaccination among health care providers [20–22]. Statements such as ‘administer with caution’ do not provide added scientific or practical information on the use of the vaccine and may cause misperceptions. Similarly, product information with many vaccines include cautionary language such as ‘if the benefits outweigh the risks’, or ‘your doctor needs to assess the benefits and potential risks of giving you the vaccine if you are pregnant’. Such statements do not resolve complex accountability issues for the vaccine but suggest a shifting of responsibility from manufacturers and regulators to healthcare providers. They may be perceived by healthcare providers as additional deterrent to recommending and administering influenza vaccine to pregnant and lactating women [23].

The study was limited to the product information available online, likely skewing the results toward high resource countries with widespread internet use. Online information in languages other than English or from websites that did not provide English translations were also not included. It is unclear from our study what information is included in non-English product information regarding use during pregnancy or lactation. Our review reveals a considerable variability in the language used in product information and show that few products reference the current public health recommendations. It could be useful to study package inserts in Russian, Mandarin, etc. to see if results are similar.

Further studies are indicated to determine the factors that contribute to low immunization coverage rates in pregnant women, including understanding by healthcare providers of the benefits to the mother, the developing fetus, and the infant in the first few months after birth [24]. The evidence resulting from such studies could help inform and optimize educational interventions targeting health care workers recommending or giving influenza vaccines to pregnant women [25,26]. Also, there are differences in influenza vaccines that could affect safety [17], and many vaccines do not specify that studies have been done in pregnant women indicating the need for additional studies to assess safety in early pregnancy.

Typically, the format and content of vaccine product information is defined by law. Manufacturers propose the wording and the information is approved by the relevant national regulatory authority. Assuming that product information must not be false or misleading and must not contain implied claims or uses for which there is inadequate evidence [27], updating product information language to include recent safety studies and reviews should be considered by manufacturers and regulators. In the absence of safety data for specific products, manufacturers and public health authorities should generate additional safety data for use of these products by pregnant women. We do not believe that there is a need for additional safety data on the use of influenza vaccines in lactating women.

Regulators from several countries in the European Union and the US Food and Drug Administration (US FDA) have started to address content issues related to package inserts (the term used by US FDA) for vaccine use in pregnant women.

WHO is working with regulators and manufacturers to support evidence-based use of vaccines during pregnancy by reviewing the wording in product information for several vaccines and is developing a guidance document to help interpret information in pregnancy subsections of product information for inactivated influenza vaccines [27]. Such complementary information may help healthcare providers understand the benefits as well as the available data on the safety of influenza vaccines during pregnancy and mothers who are breastfeeding [28]. More well-defined WHO recommendations or guidelines could conflict with national legal requirements for the format of a product information.

## Figures and Tables

**Table 1:** Summary information for influenza vaccine product information for pregnant women.

Category	Seasonal	Pandemic	Total
Pregnancy Information	65	31	96
Can be used	9 (14%)	1 (3%)	10 (10%)
Not recommended or forbidden	3 (5%)	1 (3%)	4 (4%)
Administer with caution	1 (2%)	0	1 (1%)
After first trimester	1^a^ (2%)	0	1 (1%)
Health care provider to assess	29^a^ (45%)	19 (61%)	48 (50%)
If benefits outweigh risks	1 (2%)	0	1 (1%)
If clearly needed	17 (26%)	9 (29%)	26 (27%)
No data	1 (2%)	1 (3%)	2 (2%)
Not applicable	3 (5%)	0	3 (3%)

a For those at high risk the recommendation is for any stage of pregnancy (1 in category ‘after first trimester’ and 2 in ‘health care provider to assess’).

**Table 2:** Summary information for influenza vaccine product information for women who are breastfeeding.

Category	Seasonal	Pandemic	Total
Breastfeeding Information	65	31	96
Can be used	33 (51%)	12 (39%)	45 (47%)
Not recommended	2 (3%)	1 (3%)	3 (3%)
Health care provider to assess	3 (5%)	6 (19)	9 (9%)
If benefits outweigh risks	2 (3%)	1 (3%)	3 (3%)
If necessary	0	2 (6%)	2 (2%)
Use with caution	15 (23%)	6 (19%)	21 (22%)
No data available	3 (5%)	1 (3%)	4 (4%)
NA	3 (5%)	0	3 (3%)
Breastfeeding not mentioned	4 (6%)	2 (6%)	6 (6%)
